# Life Expectancy in Chinese Cities: Spatially Varied Role of Socioeconomic Development, Population Structure, and Natural Conditions

**DOI:** 10.3390/ijerph17186597

**Published:** 2020-09-10

**Authors:** Daquan Huang, Shuimiao Yang, Tao Liu

**Affiliations:** 1 School of Geography, Faculty of Geographical Science, Beijing Normal University, No. 19, Beijing 100875, China; huangdaquan@bnu.edu.cn (D.H.); 201921051051@mail.bnu.edu.cn (S.Y.); 2 College of Urban and Environmental Sciences, Peking University, Beijing 100871, China; 3 Center for Urban Future Research, Peking University, Beijing 100871, China

**Keywords:** life expectancy, spatial analysis, geographically weighted regression, prefecture-level city, China

## Abstract

Improving life expectancy, as well as people’s health and wellbeing, is an important goal both for the Chinese government and the United Nations. Therefore, to analyze the main factors influencing life expectancy in prefecture-level cities in China, this study uses classical ordinary least-squares regression and geographical weighted regression on the data of the latest census. Moreover, regional differences induced by each influencing factor are also depicted in this study. The results demonstrate that there is significant heterogeneity and spatial positive correlation among the distribution of life expectancy in prefecture-level cities, with a generally higher life expectancy in the provincial capitals and eastern China, and lower in western China. The geographically weighted regression analysis shows that the economic development level, medical conditions, demographic structure, natural environment, and city attributes all affect the distribution of life expectancy, but that their effects have significant spatial heterogeneity. Life expectancy of the less developed areas in Western China is affected dominantly by economic development level, whereas medical services and education are of great importance in determining the life expectancy in Northern and Southern China, respectively. Thus, it is crucial to solve health problems based on local conditions, especially focusing on the improvement of health and health care in underdeveloped areas. Meanwhile, for the eastern developed areas, special attention should be paid to environmental protection in the economic process, while striving to achieve high-quality development.

## 1. Introduction

In addition to being the most important index in the evaluation of levels of health and aging, life expectancy is also a comprehensive index used to evaluate levels of economic development, education, and medical and health services [1]. The United Nations Sustainable Development Goals (2015–2030) clearly put forward various measures and methods, such as reducing the global maternal mortality, ending preventable deaths of newborns and children, reducing the death rate of various communicable and non-communicable diseases, and deaths and injuries from road traffic accidents, to ensure healthy living and promote wellbeing [2]. Therefore, how to scientifically measure life expectancy in a country or region, study its influencing factors, and formulate effective government measures to increase life expectancy and improve health and wellbeing have always been hot topics for governments and scholars [1,2,3,4,5]. Many studies have shown that natural environment; the level of economic development, education, and medical and health service; living environment; living habits; behavioral patterns; and marital status play an important role in life expectancy. At the same time, there are significant differences in the factors influencing life expectancy in various countries or regions in different developmental stages. Therefore, countries and regions need to formulate health policies according to their own specific conditions to improve life expectancy [3,4,6,7,8,9,10,11,12].

Increasing life expectancy and improving people’s health and wellbeing are also important policy objectives of the Chinese government. Since the Reform and Opening Up, life expectancy in China has greatly increased [1,13,14]. The 13th Five-Year Plan in China proposes to increase life expectancy by 1 year from 2015 to 2020 [15]. In addition, the Healthy China Action (2019–2030) explicitly takes life expectancy as the main indicator in the action plan [5]. Some empirical studies point out that China covers a large territory, with local economic development varying greatly, and there are differences in the factors influencing life expectancy in different places and at different stages of development [1,13,14]. Therefore, it is up to local governments to accurately assess life expectancy and its influencing factors, as well as to formulate effective policies to achieve the goal of increasing life expectancy. However, at present, China only publishes life expectancy in the national and provincial census years [16], and studies on the influencing factors of life expectancy only reach the provincial level, lacking research on urban and local scales, which makes it difficult to meet the needs of formulating effective policies to improve life expectancy [1,13,14].

Since the economic reform and opening up, and with the aim of bridging the gap between the natural environment and economic development at the provincial level, empowering central cities to play a leading role in regional economic development, China began to implement the “city administering counties system” as early as the 1980s [17]. After more than 30 years of development and improvement, the prefecture-level city has become an important measure for organizing China’s regional economic development and government public service [17,18,19]. In fact, education, medical and health services, and other facilities that affect life expectancy all represent the infrastructure and public service facilities at the city level [1,13,14]. Therefore, based on previous research and the data of the 6th census, this study discusses the spatial characteristics and influencing factors of life expectancy in prefecture-level cities in China. Scientific research questions of this research include: (1) how to scientifically calculate life expectancy in prefecture-level cities in China? (2) what are the factors that affect life expectancy in contemporary China? and (3) how do the dominant factors vary across vast China.

Following the introductory part, the structure of this paper is as follows: (1) The second part is the literature review, mainly covering the research progress on the influencing factors of life expectancy. (2) The third part introduces the study area, data, and methods. (3) The fourth part presents the research results. It includes the spatial characteristics of life expectancy and the factors influencing life expectancy in prefecture-level cities in China. (4) The fifth part presents the conclusions and policy implications.

## 2. Literature Review

In recent years, studying the influence of different factors on life expectancy has become a hot point of academic research [6,9,12,20,21,22,23,24,25,26,27]. In addition, scholars have been greatly interested in the influence of economic development on life expectancy. According to Richard Wilkinson’s income inequality hypothesis, the effect of economic development on health follows the law of diminishing marginal returns, and income inequality is the main factor leading to differences in life expectancy in developed countries, which further leads to social fragmentation and conflicts, relative deprivation, and long-term pressure [28]. Narrowing the income gap will improve people’s health and wellbeing [11]. Many empirical studies also show that there is no simple linear relationship between economic development level and life expectancy [29]. In developing countries, socio-economic change may have a greater impact on health improvement than technological progress [22]. The level of national income, urbanization, and other socio-economic indicators contribute mostly to a healthy life expectancy [12]. However, as the economy develops to a certain extent, living habits and behavioral patterns have a more important impact on life expectancy [28,29]. Thus, for China, some studies show that the impact of economic development level on life expectancy is similar to Richard Wilkinson’s theoretical hypothesis [28,30]. However, the difference in the economic development level between cities is far greater than that between provinces in China. Therefore, more empirical studies are needed on the impact of urban economic development on life expectancy.

The level and availability of medical and health resources are important factors affecting life expectancy. In developed and developing countries, there are significant differences in the scarce types and responsibilities of medical and health resources. Thus, in developing countries, the level of basic public health services has an important impact on life expectancy [31]. In places where health resources are insufficient, increasing preventive care and the availability of health care resources is essential to increasing life expectancy. However, in developed countries, early diagnosis of chronic diseases has an important impact on life expectancy [8]. If more equitable access to health care and health interventions could be achieved, the gap in mortality between the black and the white could be effectively reduced [32]. For a life expectancy of at 65 years of age, an empirical study finds that careful care for the elderly is essential, and life expectancy at 65 years of age is significantly related to the number of public health nurses [4]. Similarly, in China, there are differences in the level and availability of medical and health resources among different cities.

Age structure, educational level, family size, and other indicators reflecting demographic structure are important factors affecting life expectancy. Considering the proportion of the population aged 15–64, a higher proportion of those aged 0–14 indicates a higher birth rate in the areas that are generally considered more backward, and also indicates a lower proportion of the population aged >65 years. The extent of family planning can affect life expectancy in a region through its effect on the demographic structure [33]. Furthermore, educational level is an important factor affecting life expectancy [12]. It is generally believed that people with higher education tend to accept new things and new lifestyles faster, have a wider range of knowledge, learn more about nutrition, health, and self-care, and have a deeper understanding of the impact of various factors on health, which is conducive to the development of good living habits. People with a higher educational level tend to have a higher income and better quality of life, and also better living and sanitary conditions. Women with a higher educational level will be less influenced by traditional ideas. They pay close attention to fewer and better births, and child rearing will be more backed up by science, which promotes a decline in fertility and mortality rates. Another empirical study shows that in different ethnic and gender groups, the higher the educational level is, the longer the life expectancy [7]. Increased differences in the educational level lead to increased differences in life expectancy [9]. Other studies have shown that the impact of family size on life expectancy is closely related to economic development level. First, family size in developed areas is relatively small, so it is negatively correlated with life expectancy; second, with further development of the economy, the impact of family size on life expectancy gradually becomes significant [33].

In China, there are significant regional differences in the correlation between topographic relief and population distribution [34]. Topography and other natural conditions have a profound impact on human economic activities. Some empirical studies have shown that natural factors, such as topography and climate, have a significant impact on life expectancy [35,36,37]. The altitude of western China is higher than that of eastern China, with Tibet and Qinghai over 3000 m and other provinces and regions in western China around 1000 m, while most of eastern China is below 500 m. The high altitude, low temperature, and arid areas and sand in western China are not conducive to human survival [13]. However, a suitable climate, including moderate temperature and rainfall, has a positive impact on life expectancy [37,38]. Meanwhile, some studies have shown that air quality has a negative impact on life expectancy by increasing the lung cancer mortality rate [39].

Urban location, urban population size, urban administrative scale, and other attributes of cities are also important factors affecting life expectancy. Empirical studies show that these factors are closely related to regional economic development level, medical conditions, and natural environment, thus affecting life expectancy. The marginal effect of economic development on life expectancy presents a decreasing trend. Generally, eastern cities have a higher level of economic development, while the central and western cities have a lower level of economic development but a faster growth rate. Therefore, economic development plays a more significant role in improving life expectancy in underdeveloped areas [40]. Moreover, the economic development level in the western areas is generally poorer, which is not conducive to the improvement of people’s living standards and medical and health conditions [41]. Compared with developed areas, the level of urbanization has a greater impact on the difference in life expectancy among underdeveloped areas. In developed and underdeveloped areas, the quality of medical personnel and institutions may be quite different [42]. Some studies have also found that the climate environment in different regions of the east and west will lead to significant differences in life expectancy [3,40].

## 3. Study Area, Data, and Methods

We chose China, with large differences in physiographic conditions, economic development, medical conditions, as the study area to analyze the spatial variation in influencing factors of life expectancy. Specifically speaking, this paper first describes the overall spatial pattern of life expectancy, and then uses the ordinary least square (OLS) regression model to analyze the overall influencing factors of life expectancy. After that, the OLS regression analysis is conducted in four regions, respectively, to demonstrate the validity of the model and compare the results, which further illustrates the necessity of using geographically weighted regression (GWR) for a more micro-scale analysis of regional differences in factors influencing life expectancy.

### 3.1. Study Area

**Figure 1:**
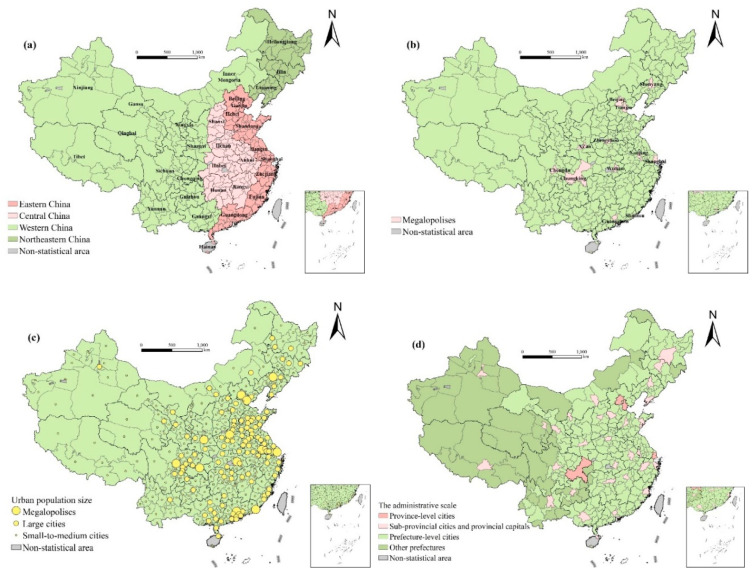
The study area: (**a**) the regional division; (**b**) megalopolises; (**c**) urban population size; (**d**) administrative level.

This study includes a total of 336 prefecture-level cities in China as research subjects. There are many cities of various types, suitable as research areas and research objects. Considering the urban population size, there are 14 megalopolises (urban population >5 million), 112 large cities (urban population 1–5 million), and 210 small-to-medium cities (urban population < 1 million). According to the urban administrative scale, there are four province-level cities directly under the central government, 32 sub-provincial cities, and provincial capitals, 250 prefecture-level cities, and 50 other prefectures. According to the regional division, there are 87 cities in eastern China, 82 cities in central China, 131 cities in western China, and 36 cities in northeastern China (Figure 1).

### 3.2. Data

The life expectancy data are derived from the average population and number of deaths of all ages from 336 prefecture-level cities, based on the 6th census. Census data are the only reliable source of information about the average population and number of deaths at the city level. This is the main reason for our selection of 2010, when the latest round of national census was conducted to do this city-level research on life expectancy. The influencing factors in the model are mainly from the 6th census, China Regional Economic Statistical Yearbook for 2011, China City Statistical Yearbook for 2011, and Statistical Yearbook of Provinces, also for 2011.

### 3.3. Calculation of Life Expectancy

The method to calculate life expectancy is to track and investigate the group of people born at the same time, record the death toll of each age group until the end of the last person’s life, and then calculate life expectancy according to the number of people living up to different ages. As it is very difficult to track the complete life cycle of a group of people born at the same time, we can often use the mortality level of different ages in the same year to replace the mortality level of the same generation at different ages in application. Then, we can calculate the average survival number of people of all ages, thus calculating the life expectancy in this year. Therefore, life expectancy is related to the mortality rate during the same period. The calculation steps of the abridged life table are as follows:

(1) Calculating age-specific mortality rate nm_x_:nm_x_ = D_X_/P_X_
(2) Calculating age-specific probability of death nq_x_:nq_x_ = 1 − $e^{-n\left(nm_{x}\right)-am^{3}{\left(nm_{x}\right)}^{2}}$, α is the empirical value, according to Reed–Merrell, α = 0.008.
(3) Calculating age-specific number of survivors l_x_ and deaths 5d_x_, assuming l_0_ = 100,000. 5d_x_ = l_x_ × 5q_x_, l_x+5_ = l_x−5_ d_x_
(4) Calculating total person-years of survival T_x_
  The values of each group aged 10 years and above were calculated from the higher age group by the following formula: T_x_ = −0.20833l_x−5_ + 2.5l_x_ + 0.20833l_x+5_ + 5$\overset{\infty }{\underset{\alpha =1}{\sum }}l_{x+5\alpha }$
(5) Calculating person-years of survival 5 L_x_:L_0_ = 0.276l_0_ + 0.724l_1_
  L_1_ = 0.410l_0_ + 0.590l_2_
  5 L_5_ = −0.003l_0_ + 2.242l_5_ + 2.761l_10_
  5 L_x_ = T_x_ − T_x+5_
(6) Calculating life expectancy $e_{x}:e_{x}=$ T_x_/l_x_

In this paper, an abridged life table was compiled using the Reed–Merrell method by the Statistical Analysis System (SAS) to calculate life expectancy in prefecture-level cities in 2010. The life expectancy of each age group was calculated according to the average population and number of deaths of different age groups. The Reed–Merrell method was first proposed in 1939 [43] and it is widely used. The first life table published publicly in China is “Abridged life table of male and female population in Beijing city area in 1950 and 1953” by Li Guangyin in 1957 [44]. Li found that the Reed–Merrell method was more in line with the characteristics of Chinese data [44]. Therefore, it is necessary to use this method by SAS to compile an abridged life table.

### 3.4. Spatial Autocorrelation

Exploratory spatial data analysis (ESDA) is a set of statistical methods for describing and visualizing the spatial distribution, identifying spatial atypical locations and spatial outliers, and aiming at detecting the pattern of spatial connections and the scope and form of spatial heterogeneity [45]. The core of exploratory spatial data analysis is the measurement of global and local spatial autocorrelation. In this paper, the global spatial autocorrelation can be used to describe the overall spatial agglomeration or dispersion characteristic of life expectancy, and the local spatial autocorrelation can be used to observe the agglomeration characteristic of life expectancy at a microscopic scale more accurately.

#### 3.4.1. Global Moran’s I index

The global Moran’s I index is usually used to study the overall trend and difference of the spatial correlation of observed variables in the whole research area, and its formula is:

(1) $$I=\frac{n}{S_{0}}\frac{\sum_{i=1}^{n}\sum_{j=1}^{n}W_{ij}\left(X_{i}-\bar{X}\right)\left(X_{j}-\bar{X}\right)}{\sum_{i=1}^{n}{\left(X_{i}-\bar{X}\right)}^{2}}$$

where n is the number of spatial units in the study area, and $X_{i},\;X_{j}$ are the observations of a space unit at point i and j; $\bar{X\;}\mathrm{is}\;\mathrm{the}\;\mathrm{mean}\;\mathrm{of}\;\mathrm{the}\;\mathrm{observations},\;W_{ij}$ is spatial weight matrix, and $S_{0}$ is sum of the spatial weight matrix.

#### 3.4.2. Local Moran’s I index

The local Moran’s I index can be used to reveal the heterogeneity between the attributes of different spatial units in the study area, and its formula is:

(2) $$I_{i}=Z_{i}\overset{n}{\underset{i=1}{\sum }}W_{ij}Z_{j}$$

where $Z_{i}$, $Z_{j}$ are the normalized value of the observations of a space unit at point i and j, and $W_{ij}$ is the spatial weight.

Local spatial autocorrelation can be studied by Moran scatter plot [46]. Areas located in the first quadrant of the Moran scatter plot have large observed values themselves, and nearby areas also have large observed values. Such areas are called high-high (HH) type areas. The areas in the second quadrant has smaller observations of its own, but the areas around have larger observations. The areas in the second quadrant are called low-high (LH) type areas, and the other quadrants follow.

### 3.5. Empirical Model

#### 3.5.1. Influencing Factors and Variable Selection

The dependent variable used in this study is life expectancy at birth in the prefecture-level cities in 2010. Considering the influencing mechanism of life expectancy mentioned above and data availability, 17 representative indicators were selected from 5 aspects—economic development level, medical conditions, demographic structure, natural environment, and city attributes—to analyze the factors influencing life expectancy.

This study uses the logarithm of per capita gross domestic product and urbanization rate in the prefecture-level cities to measure the local economic development level [12,28,30]. In terms of medical conditions, because the allocation of personnel and beds of medical institutions in China is carried out under the guidance of documents prepared by relevant health authorities, and the number of health technical personnel is highly related to the number of hospital beds, health care and the infant mortality rate are selected to measure the quality of medical and health services [8,31]. It is noteworthy that infant mortality rate is also a component of life expectancy. The influence of this factor can also reflect the contribution of this part to the overall pattern of life expectancy of a city. The logarithm of per capita health expenditure is selected to measure the government investment in medical and health services, which is conducive to solving the difficulty and high cost of obtaining medical services. Road density is used to measure the availability of medical and health services, which is crucial to improving life expectancy [32]. To reflect the influence of demographic factors on life expectancy, the proportion of the population aged ≥65 years is selected to reflect the population aging, the average years of education to reflect the educational level. At the same time, with the change of people’s notions of life and family, the family structure has also undergone great changes over the past decades in China, which are also probably very likely to influence life expectancy [12,33]. More importantly, larger size of a family indicates more resources that family members can share and enjoy and more care the elderly can get from family members, both of which are very likely to increase life expectancy. Hence, we include family size, namely, the average number of people per household in the empirical models. In view of the gender difference of life expectancy, the sex ratio is selected to control this variation [7]. Furthermore, considering the large-scale internal migration and its possible influence on life expectancy, the proportion of migrants in total population is also selected to examine the existence of this influence. In terms of the natural environment, the average annual PM2.5 concentrations were selected to reflect air quality, the logarithm of average altitude to measure terrain conditions, and the perennial mean temperature to reflect the impact of temperature on life expectancy [35,36,37]. Meanwhile, due to the differences in the economic development level, medical conditions, and natural environment, considering the characteristics of the city itself, this study selected the urban location, urban population size, and urban administrative scale to measure the impact of city attributes on life expectancy. The definitions and basic statistical characteristics of the variables are shown in Table 1.

#### 3.5.2. Geographically Weighted Regression Model

This study attempts to construct a geographically weighted regression (GWR) model of life expectancy and its influencing factors to quantitatively analyze its spatial heterogeneity. As a local model, GWR is an extension of the global spatial econometric model. It does not uniformly estimate the parameters but considers the spatial non-stationarity and performs local estimation. It is a commonly used spatial variable coefficient model that assumes that the relationship between the explained variable and explanatory variables changes with the variation of spatial position [47].

The regression model is as follows:

(3) $$Y_{i}=\beta_{0}\left(u_{i},v_{i}\right)+\sum \beta_{k}\left(u_{i},v_{i}\right)x_{ik}+\varepsilon_{i}\;i=1,2,\ldots n$$

where $\left(u_{i},v_{i}\right)$ is the spatial two-dimensional coordinate of the *i*-th sample point, and $\beta_{k}\left(u_{i},v_{i}\right)$ is the value of the continuous function $\beta_{k}\left(u,v\right)$ evaluated at point i. If there is no fluctuation in the value of $\beta_{k}\left(u_{i},v_{i}\right)$, it is proved that there is no significant spatial non-stationarity; $\varepsilon_{i}$ is the random error of point i, and the random error of different sample points is independent and identically distributed.

Moreover, the GWR model adds spatial weight to the surrounding samples in the local regression of each point, which requires the spatial weight matrix between regions. In this study, the adaptive bandwidth of the quadratic kernel function and the modified AIC (Akaike Information Criterion) are used for regression modeling. The MGWR 2.0 is used to obtain the model results and draw the coefficient distribution map. The magnitude and symbol of the estimated regional coefficient reflect the degree and direction of each variable on the life expectancy in different regions.

## 4. Results

### 4.1. Spatial Distribution Characteristics of Life Expectancy in Prefecture-Level Cities

First, the spatial autocorrelation of life expectancy and its explanatory variables were tested. The Moran’s I index and standardized Z score of global autocorrelations were calculated by GeoDa. The results show that all variables have significant spatial autocorrelation, which proves the necessity of using a spatial regression model (Table 2).

**Figure 2:**
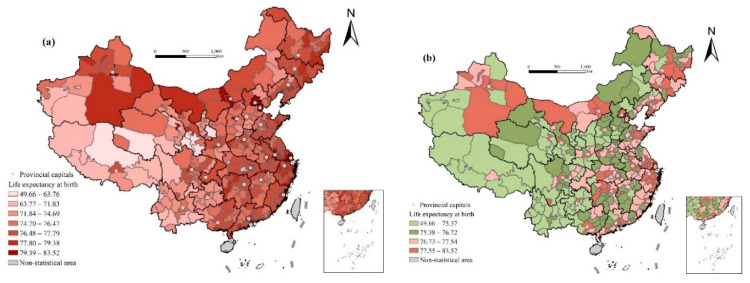
Spatial distribution of life expectancy at birth in prefecture-level cities in 2010: (**a**) the whole spatial pattern; (**b**) quartiles.

As it can be seen from Table 2, in 2010, the minimum life expectancy in a prefecture-level city in China was 49.66, which was in the Yushu Tibetan Autonomous Prefecture of Qinghai. This may be related to the massive earthquake disaster that occurred in Yushu in 2010, which caused many deaths. The maximum value is 83.52, which was in Xining of Qinghai, with a difference of 33.86 years from the minimum. The standard deviation of life expectancy in prefecture-level cities is 3.45, and the average value is 76. Moreover, as shown in Figure 2, there are significant spatial differences in the distribution of life expectancy.

Considering the distribution of life expectancy in prefecture-level cities in 2010, it can be divided into four grades based on quartiles. The first grade includes the areas with the highest life expectancy (77.55–83.52), mainly concentrated in provincial capitals and eastern coastal cities. Among them, there are 49 megalopolises and large cities, accounting for 39% of the total number, which are closely related to the advanced economy and medical conditions in these areas. The second grade includes the areas with the second highest life expectancy (76.73–77.54), mainly around provincial capitals, 78% of which are distributed in eastern China, northeast China, and central China. There are 39 megalopolises and large cities, accounting for 31% of the total. The third grade includes the areas with higher life expectancy (75.38–76.72). Among them, there are 30 megalopolises and large cities, accounting for 24% of the total. The fourth grade includes the areas with the lowest life expectancy (49.66–75.37), 82% of which are distributed in western China. Tibet, Yunnan, Guizhou, and western Sichuan in southwest China, and Ningxia, western Xinjiang, southern Gansu, southern Qinghai, and northern and southern Shaanxi in northwest China are the main gathering areas, which generally have relatively backward economic development, high altitude, and high infant mortality rate (Figure 2).

**Figure 3:**
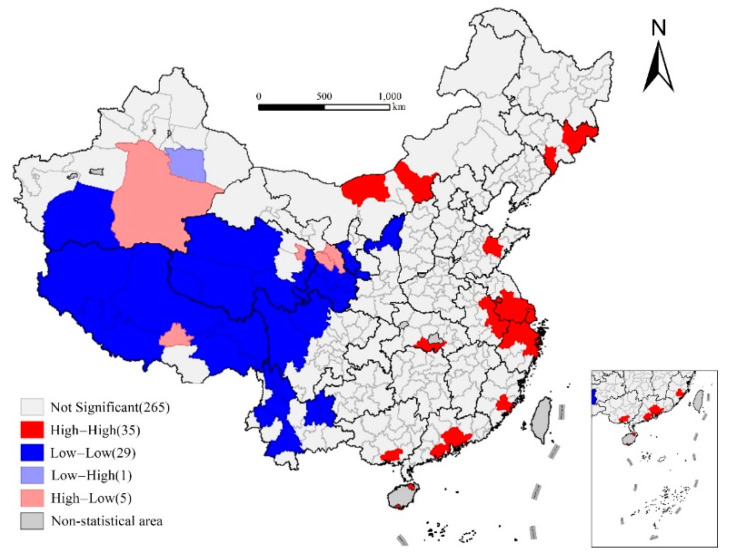
LISA Cluster Map of life expectancy at birth in prefecture-level cities in 2010.

Further cluster analysis of local indicators of spatial association (LISA) shows that the high-value agglomeration areas of life expectancy are mainly distributed in the eastern coastal areas and the central and western regions of Inner Mongolia. The central and western regions of Inner Mongolia are important regions for the implementation of the great western development strategy, of which industrialization and urbanization have developed rapidly in recent years. The low-value agglomeration areas are mainly distributed in Tibet, western Xinjiang, Qinghai, southern Gansu, western Sichuan, Yunnan, and other higher-ground and underdeveloped areas. Overall, the low value is mainly concentrated in the southwest, and the high value is mainly distributed along the eastern coastal areas. The spatial analysis at the prefecture level can reveal the intra-provincial differences in life expectancy, especially in some provinces with large internal differences in social and economic development. For example, life expectancy in northern Gansu and northern Ningxia is higher than that in the south, and life expectancy in northern and southern Shaanxi is significantly lower than that in the central plain. In addition, life expectancy in eastern Xinjiang is significantly higher than that in the west. Thus, the spatial pattern of life expectancy has obvious spatial heterogeneity, so it is difficult to predict a universal explanation model. The GWR model focused on spatial heterogeneity has more potential to provide a reasonable explanation for this phenomenon (Figure 3).

### 4.2. Analysis of Factors Influencing Life Expectancy

To investigate the factors influencing life expectancy, a global ordinary least-squares (OLS) regression analysis was first conducted in this study. Table 3 shows the OLS regression results.

There is a significant positive correlation between per capita GDP and life expectancy in prefecture-level cities, which supports the view that the economic development level played an important role in life expectancy in previous studies [22,28,29,30].

The quality of medical and health services has a significant impact on life expectancy. The number of health technical personnel per thousand has no significant effect on life expectancy. The infant mortality rate is the most influential variable with a regression coefficient of −0.336, indicating that the higher the infant mortality rate, the lower the life expectancy [48]. On the one hand, this result indicates the great contribution of infant mortality as a part of overall life expectancy to the latter. On the other hand, infant mortality rate is an important indicator reflecting the levels of health, social and economic development, and medical technology of a country or nation, and it is an especially important indicator of the maternal and child health care level, which also shows that the improvement in medical and health service quality is of great significance for health improvement.

The average years of education and average household size have a significant impact on life expectancy. The standardized regression coefficient of average years of education is positive, indicating that the higher the educational level, the better the health conditions, thus verifying the results of previous studies [7,9,12,42]. People with a higher educational level will learn more about nutrition and self-care, which is conducive to the development of good living habits. At the same time, women with higher educational levels will be less influenced by traditional ideas. They pay attention to fewer and better births, and child rearing will be more based on science, which promotes a decline in fertility and mortality rates [33,42,48,49]. The average household size has a significant negative impact on life expectancy, that is, the smaller the family size, the longer the life expectancy. Generally, family size in developed areas is relatively small, so it is negatively correlated with life expectancy [33]. At the same time, the high cost of education and living in modern society leads to the high cost of childrearing. Therefore, parents with fewer children have less economic and mental pressure, and their life expectancy is relatively long. In addition, the modern relatively perfect medical security system and higher level of pension welfare encourage older people in cities to live separately from their children, which also means the formation of a new supporting model, thus reducing intergenerational friction [49]. However, the negative association between family size and life expectancy may not be the case all across China for the great variation in socioeconomic development and healthcare conditions. Sex ratio and the proportion of migrants do not have significant effect on life expectancy in the OLS regression. On the whole, there is a negative correlation between the sex ratio and life expectancy. The higher the sex ratio, the greater the proportion of the male who generally have a lower level of average life expectancy than that of female [7]. In this sense, this negative correlation, though not significant, is reasonable. Furthermore, the influence factors of life expectancy are very likely to vary across gender, which might be an important issue to be addressed in future research when more detailed data are available.

Temperature conditions also has a significant impact on life expectancy. The standardized regression coefficient of perennial mean temperature is 0.234, which indicates that the higher the perennial mean temperature, the longer the life expectancy (Table 3). This may be due to the special natural and geographical environment of China. The perennial mean temperature is generally lower in the western regions with higher terrain and less development, while the perennial mean temperature is generally higher in the lower-lying and more developed eastern regions. However, the effect of average altitude on life expectancy is not very significant. This may be because China has a vast territory with complicated topography. Therefore, the influence of average altitude on life expectancy in different regions may vary, which finally leads to insignificant results in global OLS regression. This further illustrates the importance of local regression considering spatial heterogeneity.

OLS regression analysis is then conducted in eastern, central, western and northeastern China, respectively. The results show great variation in influencing factors of life expectancy across these four regions with different locations, policy contexts, and socioeconomic development levels (Table 4). For eastern China, the infant mortality rate, average household size, average years of education, the proportion of migrants, air quality and average altitude are all important factors of life expectancy. However, average years of education and temperature are the most important for life expectancy in northeastern China. These results further illustrate the necessity of using GWR for a more micro-scale analysis of influencing factors on life expectancy.

Table 5 shows the GWR regression results. The AICc (the modified AIC) value obtained from the fitting results is 623.047, which is smaller than that of the global regression model (647.464). The adjusted R-square value of the model increases from 0.626 (OLS) to 0.681, which indicates that the fitting degree of the model is greatly improved considering spatial heterogeneity, and the effects of these variables are unevenly distributed in space.

The range of residuals of the local regression model at the prefecture-level city is [−3.91, 2.62], of which more than 95% is in [−2.58, 2.58]. Therefore, the residual of the GWR model is randomly distributed at the 5% significance level. From the spatial distribution of the residuals, it can be seen that only four prefecture-level cities failed to pass the residual test. Furthermore, the spatial autocorrelation test of the residual shows that Moran’s I is 0.052, indicating that there is almost no spatial autocorrelation in the residual of the GWR model fitting results, and the overall effect of the model is very good. The following is the heterogeneity of the effect on life expectancy by means of the coefficient spatial distribution map of each variable, and then to discuss the underlying mechanism.

#### 4.2.1. Influence of Economic Development Level

**Figure 4:**
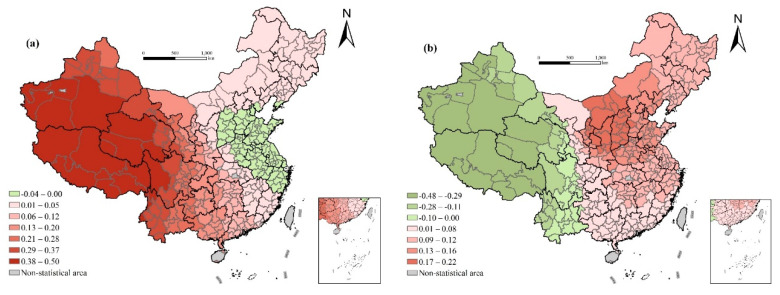
Spatial variation of regression outputs from the GWR model: (**a**) per capita GDP (ln); (**b**) urbanization rate.

Figure 4 depicts the spatial distribution of the regression coefficient of per capita GDP and urbanization rate, both of which show an obvious east–west differentiation. There is a significant positive correlation between per capita GDP and life expectancy, with the regression coefficient increasing gradually from east to west. It fully demonstrates the marginal diminishing effect of the economic development level on health [28]. In general, economic development will have a greater impact on life expectancy, but when the economy has developed to a certain extent, the growth curve of life expectancy becomes extremely gentle, and improvements must be made in medical treatment, education, living habits, and behavior patterns to effectively increase life expectancy [29]. The level of urbanization has no significant effect on life expectancy in the global OLS regression, but its effect also shows an obvious east–west differentiation. The coefficients for Yunnan, Tibet, Sichuan, Qinghai, Xinjiang, Gansu, and other less-developed western regions are generally negative, while those of other regions are generally positive, with Shaanxi and Shanxi being the highest. The offset of negative and positive effects leads to insignificant global regression. This shows that economic development does not necessarily result in an increase in life expectancy [50]. The environmental negative effects of urbanization and the society turbulence and cultural conflicts that may be brought about by the massive influx of population into cities weaken its positive effects and may have a negative impact on life expectancy.

The life expectancy of the Chinese people can continue to increase under the condition that per capita income still has great potential for growth, but considering the marginal diminishing effect of economic development level on health determines—that in order to improve life expectancy faster and more effectively, and reduce the health differences among regions—we should pay close attention to the economic development of the western underdeveloped areas and focus on the possible negative influence on life expectancy in the process of economic flow, such as air pollution, environmental damage, society turbulence, and cultural conflicts (Figure 4).

#### 4.2.2. Influence of Medical Conditions

**Figure 5:**
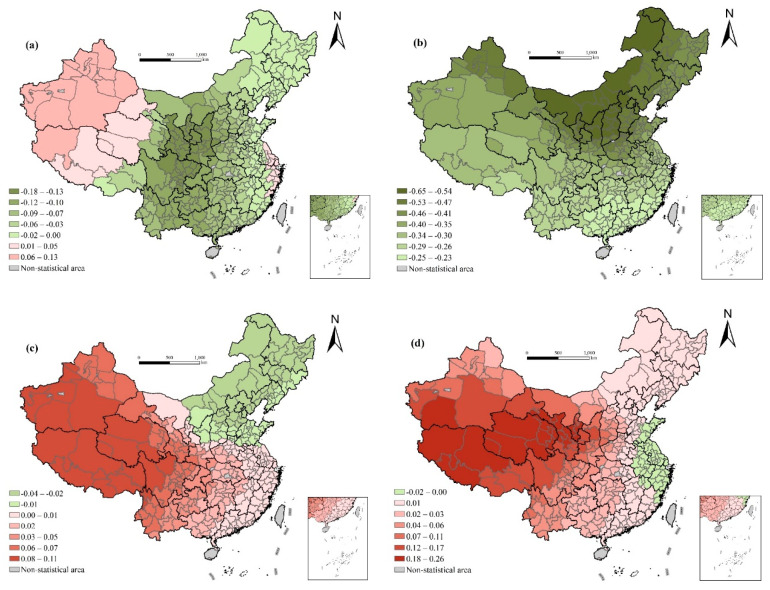
Spatial variation of regression outputs from the GWR model: (**a**) health care; (**b**) infant mortality rate; (**c**) per capita health expenditure (ln); and (**d**) road density.

The impact of health technical personnel per thousand on life expectancy is not significant in the global OLS regression, but it is negative in the local regression. This is contrary to the empirical knowledge of most people. The possible explanations for this differentiation are as follows: first, medical resources are wasted and the utilization efficiency is low due to geography, traffic conditions, population density, and economic capacity. Second, the quality of the medical staff may be poor in less developed areas [42,51]. Third, there may be a lag effect in the investment of medical and health resources on life expectancy (Figure 5).

Furthermore, there is a significant negative correlation between the infant mortality rate and life expectancy in prefecture-level cities in China. The regression coefficient gradually decreases from the south to north with some cities along the southeast coast reaching the maximum, and some cities in western Inner Mongolia reaching the minimum value, which indicates that the quality of medical and health services has a greater impact on life expectancy in northern China. This may be due to the higher socioeconomic development level, higher level of medical and health services in the south, especially in the well-developed coastal areas, and that the infant mortality rate has generally dropped. The population driving the continuous growth of life expectancy will be transferred to the elderly population aged >60 years in the future [52].

The effect of per capita expenditure for public health and road density on life expectancy is not significant in the OLS regression. In the local regression, the regression coefficient gradually increases from the east to west. This shows that the government’s investment in medical and health care has a greater impact on the improvement of health conditions in western underdeveloped areas. At the same time, in Tibet, Qinghai, southwest Xinjiang, northwest Sichuan, southern Gansu, southern Shaanxi, and other remote, higher altitude, and transportation inconvenient areas, the availability of medical and health services also has a relatively greater impact on life expectancy. The more convenient transportation is, the more conveniently residents receive get the medical and health services they need. This shows that it is not enough to focus only on the quantity of health services, but the availability of medical and health services is of great significance to the improvement of life expectancy, especially in remote mountainous areas where transportation is difficult.

#### 4.2.3. Influence of Demographic Structure

**Figure 6:**
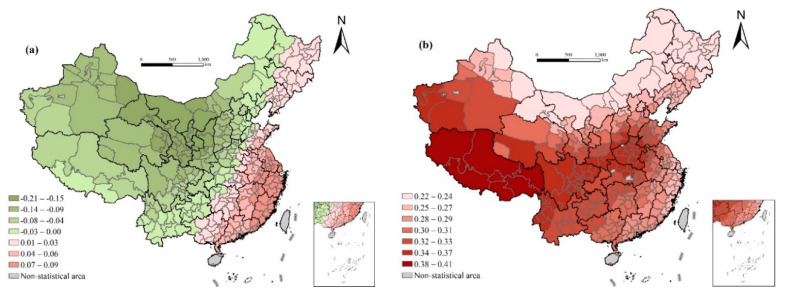

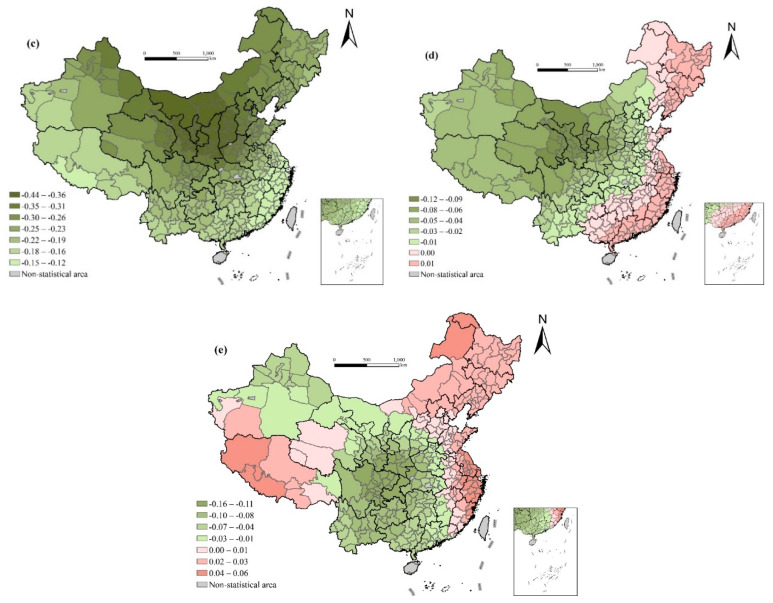
Spatial variation of regression outputs from the GWR model: (**a**) aging rate; (**b**) average years of education; (**c**) average household size; (**d**) sex ratio; and (**e**) the proportion of migrants.

There is a significant positive correlation between average years of education and life expectancy. The regression coefficient of average years of education showed a trend of gradual increase from east to west, from north to south, with the western areas (most of the prefecture-level cities in Tibet and Xinjiang) being the largest, and the coastal cities in northeast China reaching the minimum value. This shows that this segment has a greater impact on the western areas with lower educational levels, and improving the educational situation of poorer areas, especially basic education and women’s educational level, is conducive to reducing the infant mortality rate and improving health conditions (Figure 6).

The average household size shows a negative correlation with life expectancy, while the significance of this relationship is uneven across the country. From the OLS regression results above, we can see that the smaller the family size, the longer the life expectancy. A possible explanation lies in the role of family size as an important representation of regional development and modernization as described above and in previous literature [49]. The result of the local regression indicates a more complex story. The coefficient increases gradually from the central and northern regions to the southwest and southeast regions, indicating that family size has a greater impact on life expectancy in northern China. For the influence of family planning, fertility concepts, and views of marriage, these regions often have a lower level of fertility and higher level of aging than their southern counterparts. Apart from the modernization hypothesis, there exists another theory on the association between family size and life expectancy, namely the resource sharing and family care as presented in the variable section. The spatial variation of the regression result indicates that the two hypotheses are relatively balanced in southern China, while the former dominant in the north. The results also imply that the relationship between family size and life expectancy is probably more complicated than our expectation. The spatial analysis not only raises the necessity to further explore the mechanism of this relationship in future studies but also reminds us to pay more attention to regional heterogeneity in economic development and healthcare system.

The aging rate has no significant effect on life expectancy in the OLS regression, of which the regression coefficient gradually increases from the western underdeveloped areas to the well-developed coastal areas. The aging rate largely reflects the level of regional economic development. Generally, the more developed the economy is, the higher the level of aging. For the eastern coastal areas, the higher aging level reflects a higher level of economic development and life expectancy. For the northwest, southwest, and other mountainous areas, the aging rate has a negative impact on life expectancy. The possible reasons are as follows: first, theoretically, aging itself is marked by a decline in health; second, these areas are mountainous, remote, less polluted, have fresh air, and may have more long-lived elderly people, as well as a high level of aging. However, poorer economic development and medical conditions lead to higher mortality rates among low-age groups and lower life expectancy [13]. At the same time, the higher aging level leads to the economic burden caused by a rapid increase in diseases and medical service utilization demand, which has a continuous impact on the already weak economic and health development.

Although the overall effect of the sex ratio on life expectancy is not significant in the OLS regression results, its spatial variation is considerable. In the less developed western region, the negative correlation between sex ratio and life expectancy are significant, while this correlation is neglectable in central China and even positive in the coastal regions though with relatively low level of statistical significance. This spatial variation deserves in-depth investigation when more detailed data on gender difference are available. In the vast northeastern regions and Tibet, the higher the proportion of migrants, the higher life expectancy is. The possible explanation is that the population outflow in northeastern regions is serious in recent years, and the increase of the proportion of migrants means attracting more human resources, which is conducive to the development of local economy, thus improving life expectancy.

#### 4.2.4. Influence of Natural Environment

**Figure 7:**
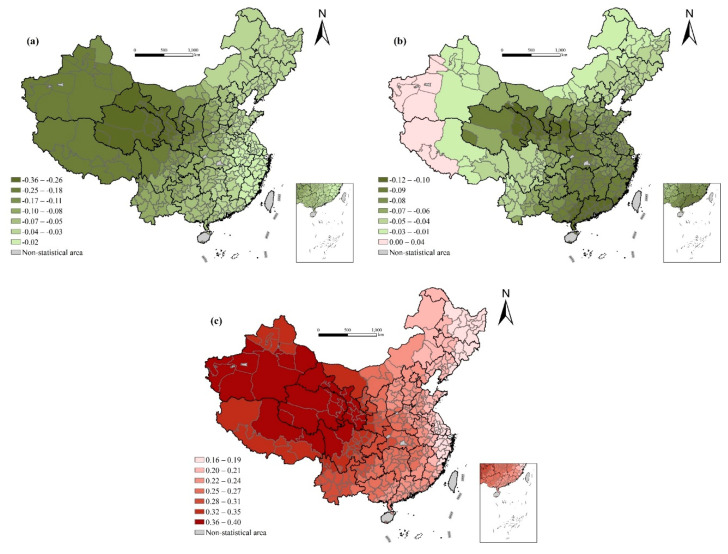
Spatial variation of regression outputs from the GWR model: (**a**) average annual PM2.5 concentration; (**b**) average altitude (ln); and (**c**) perennial mean temperature.

Overall, there is a significant negative correlation between air quality and life expectancy in most areas [39]. The regression coefficient decreases gradually from the east to west, which indicates that air quality has a greater impact on life expectancy in western areas. This may be because the air quality in the west is better than that in the east now, but the economy is in the stage of rapid development in western China, which may cause great environmental pollution and have a greater negative effect on health at a certain stage. Overall, there is a positive correlation between temperature and life expectancy. The regression coefficient gradually decreases from the west to east, indicating that the influence of temperature on life expectancy in the west is greater than that in the east (Figure 7).

Overall, the impact of average altitude on life expectancy is negative, and the regression coefficient gradually decreases from the northwest to southeast. For most prefecture-level cities in the southeast regions, average altitude has a significant negative impact on life expectancy. However, in the global OLS regression, the influence of average altitude on life expectancy is not significant.

#### 4.2.5. Influence of City Attributes

**Figure 8:**
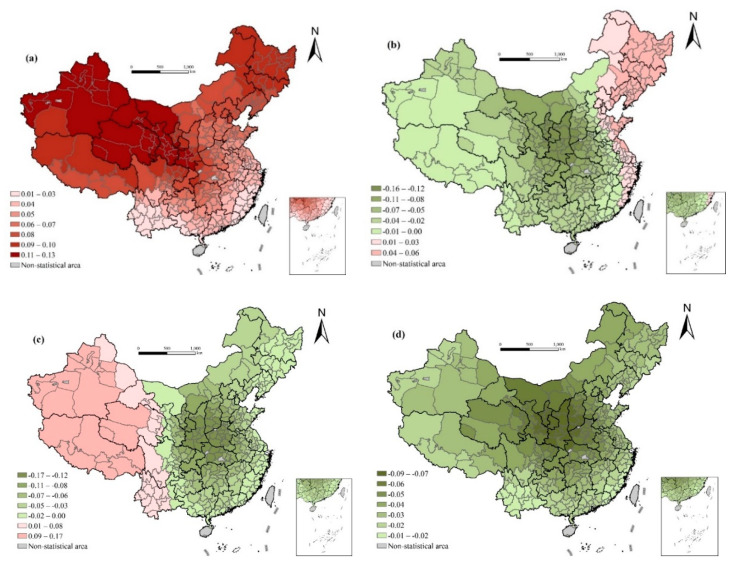
Spatial variation of regression outputs from the GWR model: (**a**) urban population size (ln); (**b**) prefecture-level cities; (**c**) sub-provincial cities and provincial capitals; and (**d**) province-level cities.

Overall, the larger the urban size, the higher the life expectancy. The regression coefficient gradually decreases from the central north to the southwest and southeast. Generally, larger cities mean higher levels of economic development, better medical and health services, and higher educational level, which is beneficial to the improvement of life expectancy (Figure 8).

As shown in Figure 8, in northeastern China, life expectancy in prefecture-level cities is higher than that in other prefectures. Life expectancy in prefecture-level cities is lower than that of other prefectures in other regions. The influence of sub-provincial cities and provincial capitals on life expectancy shows an obvious east–west differentiation, with negative effects in eastern regions and positive effects in central and western regions. This shows that life expectancy in sub-provincial cities and provincial capitals in the east is lower than that of other prefectures, but in the west, life expectancy in sub-provincial cities and provincial capitals is higher than in other prefectures. Life expectancy in province-level cities is lower than in other prefectures.

This shows that for the less developed areas, life expectancy in cities with higher administrative ranks is higher than in cities with lower administrative ranks, and for the more developed areas, life expectancy in cities with higher administrative ranks is lower than in cities with lower administrative ranks. A possible explanation is that in the eastern developed areas, with the continuous development of industrialization, many cities have seen a sharp decline in green space and even serious industrial wastewater and gas pollution. Along with the improvement of people’s living standards, the quality of living environment has declined, which is a factor that appears with social development to affect people’s quality of life, and thus may affect life expectancy [49]. At the same time, a fast-paced lifestyle may bring great pressure to people and affect their physical and mental health. However, for the underdeveloped areas, the main factors restricting life expectancy are still economic development and medical conditions, and there is still much room for economic development, which can greatly improve health.

### 4.3. Spatial Variation of Dominant Factors

**Figure 9:**
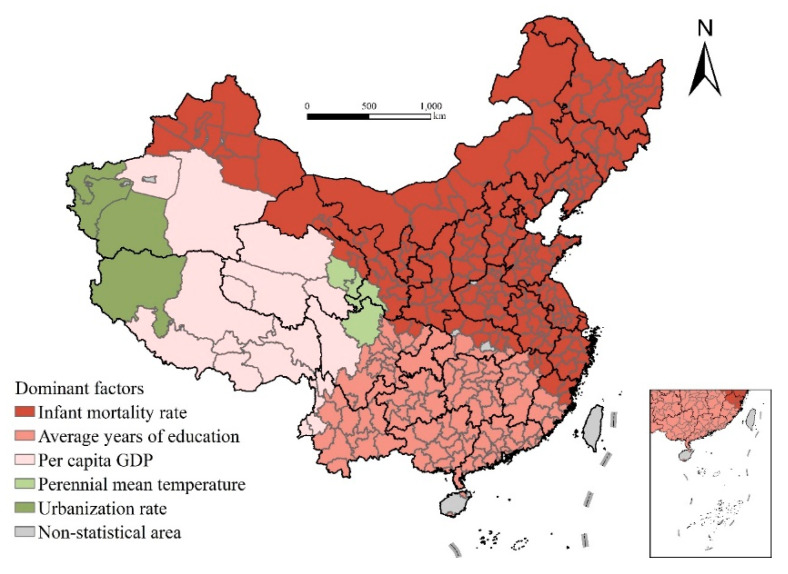
Spatial distribution of the dominant factors affecting life expectancy at birth.

The GWR model with local estimation can be used to compare the differences in the dominant factors of life expectancy. Figure 9 shows the factors with the largest absolute value of the standardization coefficient in the estimation results in the prefecture-level cities and finds two obvious characteristics. First, the differences in the leading factors of life expectancy in different regions are very obvious, which again confirms the heterogeneity of influencing factors in this study and the applicability and advantages of the GWR model. Second, the regional spatial connectivity characteristics dominated by each factor are obvious, which is closely related to the spatial continuity of geographical and cultural background factors, also explaining the rationality of the results (Figure 9).

Specifically, economic development level dominates life expectancy in Tibet, Southern Xinjiang, Qinghai, Western Sichuan, and other places because the economic development of these areas is relatively backward, and there is still much room for economic development to improve life expectancy. The leading influence of medical conditions is mainly reflected in northeast and north China, Gansu, Ningxia, Shaanxi, Henan, Shandong, Jiangsu, Zhejiang, Hubei, and other places, and the main factor is infant mortality rate. Educational level has a greater impact on the distribution of life expectancy in southern China.

## 5. Conclusions and Policy Implications

Based on the data of the 6th census from 2010, this study analyzes the spatial distribution characteristics of life expectancy and the spatial heterogeneity of its influencing factors. Although economic development level, medical conditions, demographic structure, natural environment, and other factors will affect life expectancy, the GWR model results show that the influence of these factors has significant spatial differentiation.

Economic development is an important factor affecting life expectancy. Effects of the economic development level on health follow the law of marginal decline [28]. Under the condition that per capita income still has great growth potential, life expectancy can continue to increase. Socioeconomic changes may have a greater impact on health improvement, especially in underdeveloped areas [22]. At the same time, economic development to a certain degree may bring about negative environmental effects, and there is an inverted U-shaped relationship between economic development and environmental quality.

Among the related factors of medical conditions, in general, an increase in the number of medical and health services will have a positive impact on life expectancy, especially in areas where medical resources are insufficient. Increasing the quantity of medical resources is crucial for improving life expectancy. However, at the same time, it is also affected by the quality, utilization level, and availability of medical and health services in prefecture-level cities. The infant mortality rate reflects the level of medical and health technology, which has a significant negative impact on life expectancy. Considering the spatial heterogeneity, we can find that the impact of this rate varies across different regions. Although in the global OLS regression the per capita expenditure for public health and road density have no significant effect on life expectancy, in the local regression it is found that increasing government investment in medical and health services can greatly improve life expectancy in western underdeveloped areas, and the improvement of traffic conditions in western mountainous areas can improve life expectancy by enhancing the availability of medical and health services.

The average educational level and family size have a significant impact on life expectancy, and the degree of influence is different in different regions. The average years of education has a greater impact on the western regions with lower educational levels. Improving the education status of underdeveloped areas, especially basic education and women’s educational level, is conducive to reducing the infant mortality rate and improving the health level. Family size has a significant negative correlation with life expectancy, but the degree of influence varies across regions. We also find that in the global OLS regression the aging level has no significant impact on life expectancy, but in the local regression the aging level in different regions has different degrees of impact. This is especially true in recent years, when the infant mortality rate is generally low, and the elderly play an increasingly important role.

We also find that in the global OLS regression, perennial mean temperature has significant positive effects on life expectancy. However, when considering the spatial heterogeneity of the effects, the impact model will be much more complex. The impact effect is positive in some areas and negative in others, which is related to the local natural environment and economic development.

There are great differences in life expectancy in cities with different locations, urban scales, and administrative levels. In the local regression analysis, it was found that for the less developed areas, life expectancy in cities with higher administrative ranks is higher than in cities with lower administrative ranks. However, for more developed areas, life expectancy in cities with higher administrative ranks is lower than in cities with lower administrative ranks. This reminds us that appropriate measures should be taken to improve life expectancy in underdeveloped areas and the more developed areas according to the local conditions, so as to increase life expectancy most efficiently and reduce regional health differences.

To sum up, economic development dominates the distribution of life expectancy in southwest and northwest China, whereas medical conditions largely determine the regional differences in life expectancy in northeast and northern China, Ningxia, Shaanxi, and other surrounding areas. Educational level has a great impact on the distribution of life expectancy in southern China.

Therefore, to successfully accomplish the “Healthy China 2030” plan and the United Nations Sustainable Development Goals to improve life expectancy, measures should be adjusted to local conditions to solve health problems, especially focusing on promoting the improvement of health conditions in underdeveloped areas. First, we should vigorously develop the economy of such areas, pay much attention to the quality level of economic development, strive to achieve high-level coordinated development, and focus on the negative environmental effects that may be caused in the process of economic development. Second, we should not only pay attention to the quantity of medical and health services, but also pay more attention to the quality, utilization level, and availability of medical and health services. Especially for remote mountainous areas with inconvenient transportation, we should strengthen the construction of transportation facilities. At the same time, we should increase government investment in medical and health care, improve the medical security system, and do our best to improve the problem of difficult and expensive medical treatment. Third, we should pay close attention to the situation with education in underdeveloped areas, striving to realize universal primary education for all and improve women’s educational level. Finally, we should pay attention to the health problems of the elderly, improve their medical and health care level, and vigorously reduce their mortality rate. We should seize the opportunity for the development of the western region to strengthen the population and family planning in the western provinces, to reduce the fertility rate, to change the traditional concepts, thus reducing family size.

With the continuous development of the economy, the environment, living habits, and behavioral patterns have gradually become new important factors affecting life expectancy [28,29]. In particular, in the eastern industrial developed areas, in the process of developing the economy the government and enterprises should pay special attention to the relationship between the cost-effectiveness of economy and the environment, considering policy choices from a long-term perspective [49]. At the same time, encouraging more positive and healthy living habits and behaviors is also important.

## Figures and Tables

**Table 1:** Definitions and descriptive statistics of variables.

Factors	Variables	Mean	Std. Dev.	Min	Max
*Dependent Variables*					
	Life expectancy	76	3.45	49.66	83.52
*Independent Variables*					
Economic development	Per capita GDP (ln)	10.16	0.64	8.55	11.84
	Urbanization rate	46.51%	17.13%	12.69%	100.00%
Medical conditions	Health care	4.33	1.38	1.3	9.61
	Infant mortality rate	0.51%	0.60%	0.02%	6.75%
	Per capita health expenditure (ln)	5.78	0.37	4.17	7.27
	Road density	90.99	81.11	0.43	1156.65
Demographic structure	Aging rate	8.63%	1.93%	1.79%	16.50%
	Average years of education	8.75	1.11	3.47	11.71
	Average household size	3.18	0.46	2.11	5.55
	Sex ratio	105.49	4.22	89.65	132.58
	The proportion of migrants	4.74%	8.13%	0.11%	64.87%
Natural environment	Average annual PM2.5 concentration	33.89	18.13	2.09	74.79
	Average altitude (ln)	5.77	1.59	0.26	8.53
	Perennial mean temperature	12.96	6.56	−3.52	25.54
City attributes	Urban population size (ln)	4.3	0.98	0.78	7.34
	The city located in Central China	CC = 1 (frequency: 82), else = 0			
	The city located in Northeastern China	NC = 1 (36), else = 0			
	The city located in Eastern China	EC = 1 (87), else = 0			
	Prefecture-level cities	PREC = 1 (250), else = 0			
	Sub-provincial cities and provincial capitals	SUB_PRO/PRO = 1 (32), else = 0			
	Province-level cities	PROC = 1 (4), else = 0			

**Table 2:** Global spatial autocorrelation analysis results.

Variables	Moran’s I	Z(I)	P
Life expectancy	0.424	12.535	0.001
Per capita GDP	0.396	11.961	0.001
Urbanization rate	0.402	12.107	0.001
Health care	0.292	8.627	0.001
Infant mortality rate	0.434	13.48	0.001
Per capita health expenditure	0.402	11.942	0.001
Road density	0.245	8.962	0.001
Aging rate	0.644	19.003	0.001
Average years of education	0.533	15.784	0.001
Average household size	0.572	16.677	0.001
Sex ratio	0.425	12.233	0.001
The proportion of migrants	0.572	17.165	0.001
Average annual PM2.5 concentration	0.894	26.035	0.001
Average altitude	0.905	27.102	0.001
Perennial mean temperature	0.933	26.908	0.001
Urban population size	0.086	2.742	0.013
Prefecture-level cities	0.276	8.136	0.001
Sub-provincial cities and provincial capitals	−0.081	−2.218	0.007
Province-level cities	0.087	2.814	0.032

**Table 3:** OLS regression results.

Variables	Standardized Coefficient		
	Model 1	Model 2	Model 3
Per capita GDP (ln)		0.111 *	0.162 **
		(0.341)	(0.382)
Urbanization rate		0.002	−0.026
		(0.014)	(0.015)
Health care		0.012	−0.004
		(0.155)	(0.164)
Infant mortality rate		−0.335 ***	−0.336 ***
		(0.253)	(0.263)
Per capita health expenditure (ln)		0.007	0.007
		(0.443)	(0.499)
Road density		−0.004	0.001
		(0.002)	(0.002)
Aging rate		0.013	0.029
		(0.106)	(0.112)
Average years of education		0.290 ***	0.272 ***
		(0.219)	(0.224)
Average household size		−0.204 ***	−0.196 ***
		(0.473)	(0.488)
Sex ratio		−0.004	−0.014
		(0.038)	(0.040)
The proportion of migrants		−0.027	0.010
		(0.024)	(0.027)
Average annual PM2.5 concentration		−0.095	−0.117
		(0.013)	(0.014)
Average altitude (ln)		−0.081	−0.073
		(0.137)	(0.156)
Perennial mean temperature		0.208 ***	0.234 ***
		(0.026)	(0.033)
Urban population size (ln)		0.014	0.039
		(0.197)	(0.238)
The city located in Central China	0.195 ***		0.062
	(0.440)		(0.396)
The city located in Northeastern China	0.173 ***		0.045
	(0.564)		(0.626)
The city located in Eastern China	0.260 ***		−0.035
	(0.438)		(0.460)
Prefecture-level cities	0.384 ***		−0.044
	(0.503)		(0.487)
Sub-provincial cities and provincial capitals	0.452 ***		−0.028
	(0.688)		(0.75)
Province-level cities	0.177 ***		−0.020
	(1.534)		(1.458)
Constant	72.053 ***	67.514 ***	65.54 ***
	(0.410)	(6.120)	(6.597)
Adj-R-squared	0.298	0.631	0.630
F	24.755	39.173	28.123

Notes: Standard error listed in parentheses. *** *p* < 0.01, ** *p* < 0.05, * *p* < 0.1.

**Table 4:** Results of OLS regression by region.

Variables	Standardized Coefficient			
	Eastern	Central	Western	Northeastern
Per capita GDP (ln)	0.057	0.269	0.330 **	0.272
	(−0.324)	(−0.427)	(−1.038)	(−0.667)
Urbanization rate	−0.026	0.103	−0.182	0.558
	(−0.009)	(−0.026)	(−0.042)	(−0.038)
Health care	0.183	0.236	0.021	−0.368
	(−0.132)	(−0.185)	(−0.322)	(−0.415)
Infant mortality rate	−0.260 ***	−0.106	−0.305 ***	−0.001
	(−0.844)	(−0.480)	(−0.454)	(−2.467)
Per capita health expenditure (ln)	−0.116	−0.004	0.016	0.535 *
	(−0.330)	(−0.542)	(−1.120)	(−1.509)
Road density	0.062	−0.325 **	0.035	−0.904 *
	(−0.002)	(−0.004)	(−0.003)	(−0.02)
Aging rate	0.177	0.605 ***	−0.091	0.572 *
	(−0.084)	(−0.117)	(−0.285)	(−0.227)
Average years of education	0.230 **	0.179 ***	0.350 ***	0.259 ***
	(−0.198)	(−0.290)	(−0.462)	(−0.773)
Average household size	−0.323 ***	0.228 ***	−0.142 *	0.749
	(−0.357)	(−0.638)	(−1.163)	(−2.058)
Sex ratio	0.046	0.079	−0.094	0.027
	(−0.029)	(−0.036)	(−0.086)	(−0.16)
The proportion of migrants	0.302 **	0.123	0.023	−0.065
	(−0.016)	(−0.170)	(−0.109)	(−0.131)
Average annual PM2.5 concentration	−0.282 **	0.605 **	−0.045	0.23
	(−0.009)	(−0.023)	(−0.047)	(−0.048)
Average altitude (ln)	−0.194 * *	0.095	0.041	−0.296
	(−0.097)	(−0.266)	(−0.557)	(−0.272)
Perennial mean temperature	0.172 *	−0.009 ***	0.302 ***	0.249 ***
	(−0.032)	(−0.05)	(−0.079)	(−0.149)
Constant	75.061 ***	55.288 ***	55.324 ***	35.268
	(−5.950)	(−7.603)	(−16.988)	(−22.891)
Adj-R-squared	0.749	0.49	0.569	0.182
F	19.326	6.555	13.246	1.555

Notes: Standard error listed in parentheses. *** *p* < 0.01, ** *p* < 0.05, * *p* < 0.1.

**Table 5:** Descriptive analysis of the GWR model regression coefficient.

Variables	Min	Lower Quartile	Median	Upper Quartile	Max	Average
Per capita GDP (ln)	−0.040	−0.003	0.039	0.185	0.503	0.102
Urbanization rate	−0.479	0.049	0.095	0.122	0.218	0.052
Health care	−0.182	−0.102	−0.058	−0.017	0.134	−0.058
Infant mortality rate	−0.648	−0.460	−0.354	−0.280	−0.232	−0.375
Per capita health expenditure (ln)	−0.038	−0.012	0.005	0.030	0.112	0.013
Road density	−0.018	−0.001	0.009	0.046	0.256	0.035
Aging rate	−0.207	−0.067	−0.005	0.034	0.088	−0.023
Average years of education	0.215	0.267	0.299	0.330	0.414	0.298
Average household size	−0.436	−0.294	−0.236	−0.181	−0.116	−0.246
Sex ratio	−0.117	−0.041	−0.010	0.003	0.011	−0.023
The proportion of migrants	−0.160	−0.066	−0.023	0.019	0.062	−0.029
Average annual PM2.5 concentration	−0.361	−0.104	−0.059	−0.041	−0.017	−0.090
Average altitude (ln)	−0.124	−0.094	−0.081	−0.053	0.040	−0.074
Perennial mean temperature	0.163	0.213	0.237	0.288	0.397	0.254
Urban population size (ln)	0.014	0.040	0.059	0.080	0.130	0.063
Prefecture-level cities	−0.160	−0.059	−0.029	0.003	0.056	−0.032
Sub-provincial cities and provincial capitals	−0.173	−0.078	−0.040	−0.023	0.166	−0.042
Province-level cities	−0.087	−0.053	−0.038	−0.031	−0.016	−0.043
Intercept	−0.087	−0.063	−0.047	−0.038	0.033	−0.046
